# Influenza virus genotype to phenotype predictions through machine learning: a systematic review: Computational Prediction of Influenza Phenotype

**DOI:** 10.1080/22221751.2021.1978824

**Published:** 2021-09-23

**Authors:** Laura K. Borkenhagen, Martin W. Allen, Jonathan A. Runstadler

**Affiliations:** a Department of Infectious Disease and Global Health, Cummings School of Veterinary Medicine, Tufts University, North Grafton, MA, USA; b Department of Computer Science, School of Engineering, Tufts University, Medford, MA, USA

**Keywords:** Influenza virus, machine learning, prediction, phenotype, classification

## Abstract

Background: There is great interest in understanding the viral genomic predictors of phenotypic traits that allow influenza A viruses to adapt to or become more virulent in different hosts. Machine learning techniques have demonstrated promise in addressing this critical need for other pathogens because the underlying algorithms are especially well equipped to uncover complex patterns in large datasets and produce generalizable predictions for new data. As the body of research where these techniques are applied for influenza A virus phenotype prediction continues to grow, it is useful to consider the strengths and weaknesses of these approaches to understand what has prevented these models from seeing widespread use by surveillance laboratories and to identify gaps that are underexplored with this technology. Methods and Results: We present a systematic review of English literature published through 15 April 2021 of studies employing machine learning methods to generate predictions of influenza A virus phenotypes from genomic or proteomic input. Forty-nine studies were included in this review, spanning the topics of host discrimination, human adaptability, subtype and clade assignment, pandemic lineage assignment, characteristics of infection, and antiviral drug resistance. Conclusions: Our findings suggest that biases in model design and a dearth of wet laboratory follow-up may explain why these models often go underused. We, therefore, offer guidance to overcome these limitations, aid in improving predictive models of previously studied influenza A virus phenotypes, and extend those models to unexplored phenotypes in the ultimate pursuit of tools to enable the characterization of virus isolates across surveillance laboratories.

## Introduction

Influenza A viruses (IAV) continue to be a public health and economic burden globally as well as a risk for emerging pandemic disease. Improved prediction of IAV phenotypes would aid both pandemic preparedness and mitigation of seasonal influenza disease. Accordingly, there is wide interest in understanding the viral genomic or proteomic predictors of phenotypic traits that make IAV adaptable to or more virulent in certain hosts. Machine learning techniques have demonstrated promise in generating such predictions for other pathogens [1] because machine learning algorithms are especially well equipped to uncover complex patterns in large datasets and produce generalizable predictions for new data. There are numerous facets of understanding IAV phenotypic traits where these methods can be applied, including understanding predictors that influence host tropism, interspecies transmission, virulence, and drug resistance among others. As the body of research where these techniques are applied for IAV phenotype prediction continues to grow, it is useful to consider the strengths and weaknesses of different approaches and apply this knowledge to new studies. This review discusses and compares studies where machine learning techniques have been used to predict phenotypic characteristics of IAVs and identifies areas for the follow-up to the work already performed, as well as gaps that are underexplored with this technology.

## Methods

**Figure 1.**
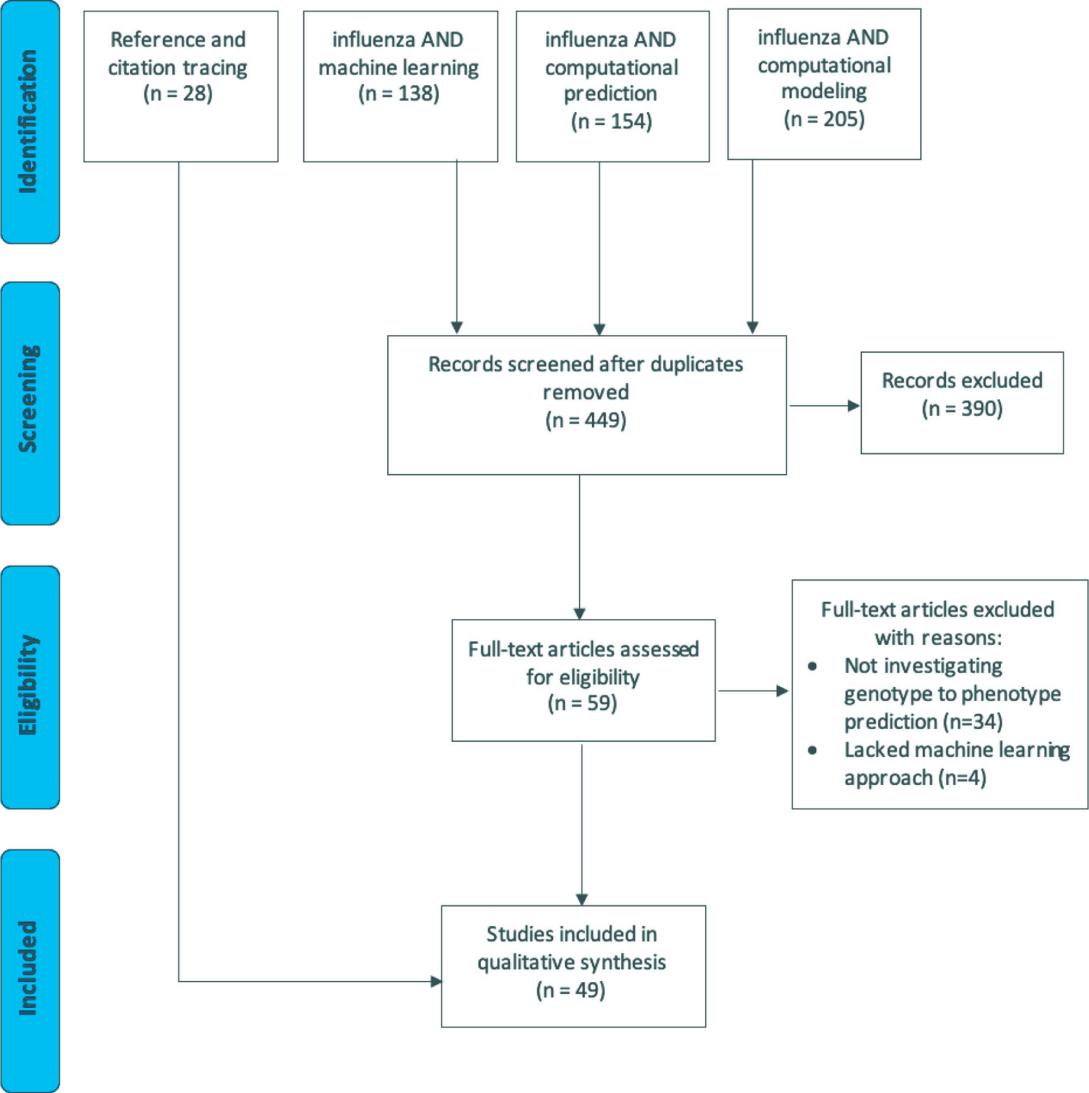
PRISMA flow diagram of the article screening process for a systematic review of influenza genotype to phenotype prediction studies employing a machine learning approach. There were 449 articles captured from 3 PubMed database searches; 28 articles were identified through tracing the references in and articles citing the full-text reviewed articles. A total of 49 articles were included in the final review.

A literature search for articles applying machine learning techniques in IAV research was initially conducted on 19 May 2020. This search followed the Preferred Reporting Items for Systematic Reviews and Meta-Analyses guidelines, though it is not a registered review [2]. The search strings “influenza AND machine learning,” “influenza AND computational prediction,” and “influenza AND computational modeling” were entered into PubMed with respective 138, 154, and 205 search results. Of the 497 total references obtained in the search, 48 duplicates were removed. The titles and abstracts of the remaining 449 articles were screened in duplicate (Figure 1). Articles were excluded from full-text review if they were non-primary sources, not available in English, or irrelevant to the review. Studies were deemed relevant to this review if machine learning approaches were employed to understand IAV phenotypic traits given viral genomic, proteomic, or proteome-derived input data. This review does not include studies that aimed to predict IAV antigenic sites, due to the host-specific nature of this topic. Given the high number of specific algorithms that could be used to generate such predictions and their other broad uses beyond machine learning, tracing of references and articles citing the included reports according to Google Scholar (both from the original search and articles previously selected through reference or citation tracing) was also performed to ensure inclusion of relevant studies not captured in the original search. The references and citations were filtered by title and traced references meeting the above criteria identified through 15 April 2021 were included in the qualitative synthesis.

## Results

### Studies included for analysis

**Figure 2.**
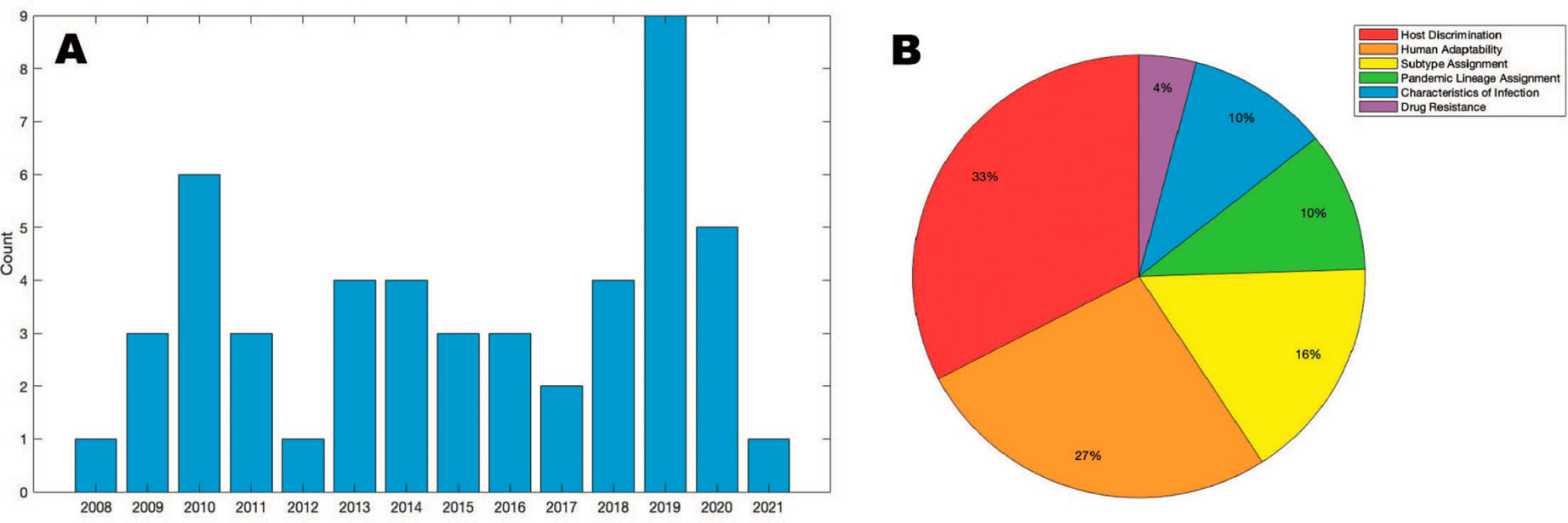
Studies employing machine learning techniques to generate influenza genotype to phenotype predictions. Studies are shown over (a) time and (b) categorization. Generated with MATLAB R2020b (The MathWorks, Inc., Natick, MA).

After the title and abstract review of references obtained through Pubmed search, 59 articles were selected for full text review; 38 articles were removed after full text review with reasons given in Figure 1. The remaining 21 articles and an additional 28 articles identified through tracing are described in Table S1. These 49 articles were separated into the following categories: host discrimination, human adaptability, subtype and clade assignment, pandemic lineage assignment, characteristics of infection, and antiviral drug resistance. Studies in this vein first appeared surrounding the 2009 H1N1 pandemic, and although interest in this area waned thereafter, there has been a clear resurgence in recent years (Figure 2).

### Method selection in analysed studies –

#### Data preprocessing

The genomic and proteomic sequence data used in these studies were extracted from one or more of the following databases in order of decreasing frequency of use: National Center for Biotechnology Information [3], Influenza Research Database (IRD) [4], Global Initiative on Sharing All Influenza Data [5], National Institutes of Health, UniProt [6], World Health Organization. The simplest and most common preprocessing approaches implemented in the reviewed studies used numerically coded amino acid or nucleotide sequences (Figure S1A and B). Many studies used composition scores. For nucleotide sequences, this consisted of mono- or di-nucleotide composition; an example of the possible utility of a dinucleotide composition score would be outcomes predicted by GC content-related antiviral immune responses (Figure S1C). For amino acids sequences, physiochemical properties such as polarity, polarizability, net charge, normalized van der Waals volume, hydrophobicity, secondary structure, and solvent accessibility were scored in terms of composition, distribution, or transition. Other studies generated amino acid matrices coding the presence of each amino acid at each position in the sequence (one-hot encoding; Figure S1D). Other unique approaches taken by Xu et al. and Kincaid used Word2Vec and N-gram methods, respectively, on amino acid sequences [7,8]. Feature reduction was often used prior to model development. This was done through feature selection, whereby statistical measures or preliminary training of machine learning algorithms are performed to identify the features most important for generating label predictions, followed by reducing the training and testing datasets to only those features before training the final model. Feature extraction, where the original features are transformed into a smaller set of new features, was also used; an example of this approach is principal component analysis (PCA).

#### Algorithm selection

Nearly all of the studies captured in this review used supervised learning, where the input samples are labelled, and machine learning techniques are used to find patterns in the sample features associated with the labels. One study used unsupervised learning, which uses machine learning to find patterns in unlabelled data.

The machine learning algorithms that tended to achieve better performance metrics in the reviewed studies were random forest (RF) algorithms [8–29] and support vector machines (SVMs) [7,8,16,19–22,24,25,30–38]. These algorithms are well suited for high dimensional data due to their use of subsets of the entire given training dataset to generate label predictions; as such, these algorithms are less reliant on a large training set and are less susceptible to outliers. Neural networks (NNs) [8,19,20,23,25,29,39–46] were also employed with relative success; they are well suited to learn complex feature interactions if the training dataset is sufficiently large. Other techniques employed with varying success included decision trees [24,25,29,31,38,40,45–51], Naïve Bayes [8,16,17,21,24,25,28,33,38], K-nearest neighbours [17,19,21–23,32,33,38], classification based on association rules [47,48,50,52,53], ADABOOST [28], logistic regression [19,29,54], rotation forest [16], gradient-boosted regression trees [20], hierarchical clustering [55], and decision-rule based methods like RIPPER/JRip [27,53], OneR [27], and PART [27].

**Figure 3.**
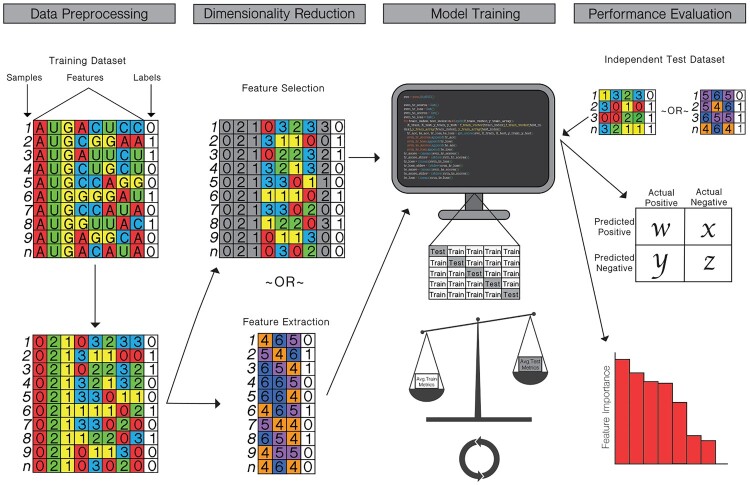
Common pipeline for training and testing machine learning classifiers to generate influenza genotype to phenotype predictions. (*Data Preprocessing*) sequence data is converted into a usable feature set and labelled. (*Dimensionality Reduction*) optionally, high dimensional data is distilled either through feature selection, in which features with low correlation or low importance for determining classification are removed, or through feature extraction, in which features are transformed into a lower dimensional plane. (*Model Training*) the machine learning classifier is trained using a K-fold cross-validation or leave-one-out analysis, in which different subsets of the data are used for training and testing the model. The average performance metrics for the training and testing folds are compared to strike a balance between overfitting and accurate model performance. (*Performance Evaluation*) the model is tested against an independent testing dataset with known labels. Performance metrics are produced, and, in some cases, the importance of specific features may be evaluated.

Testing of supervised models commonly involves K-fold cross-validation, leave-one-out validations, or use of independent test datasets. These validations help to prevent overfitting of the training data and evaluate model performance. Common metrics used to evaluate model performance on either test-folds or independent test sets include accuracy, area under the receiver operating curve (AUC), sensitivity, specificity, Matthews correlation coefficient (MCC), precision, prediction error, feature support, and feature confidence. A graphical depiction of the machine learning pipeline is presented in Figure 3, and brief descriptions of the most commonly used techniques for dimensionality reduction, modeling, and evaluation by studies in this review are in Table S2.

### Phenotypic traits examined in published studies –

#### Host discrimination

The most frequently studied phenotype in these studies was host tropism. Eleven of these studies focused on determinants of avian, human, and swine host tropism. Seven studies evaluated host prediction of their respective models trained with sequences from multiple IAV proteins [7,14,15,26,39,40,48]. Among these, Attaluri et al. and Shaltout et al. developed models that produced host predictions at or above a test accuracy of 0.98 [39,40]; however, their predictions applied only to certain HA subtypes (Attaluri et al. used subtypes H1, H3, and H5, and Shaltout et al. used H1 only), limiting the usability of such models. Xu et al. and Kwon et al. achieved only slightly lower accuracy (0.96) with models that generated predictions across all subtypes [7,17].

Three studies generated models for avian, human, and swine host predictions based on HA sequence alone [19,47,52]. The highest accuracies were achieved through a decision tree developed by ElHefnawi and Sherif, which produced test scores ranging between 0.912 and 1.000 depending on the host and subtype [47]. Yin and colleagues developed a similar RF model [19] that was later extended to all IAV proteins to estimate the probability that the sequence arose from a reassortment event [18]. Test accuracy of host prediction for each protein ranged between 0.865 and 0.965, and the model correctly identified 86% of known reassortants.

Three studies looked at avian and human host prediction [11,16,30]. Of these, the best performance was achieved by Eng et al. [11]. Predictions were generated using a RF model trained with amino acid properties. Their final model produced a test accuracy of 0.9983, sensitivity of 0.998, specificity of 1.00, AUC of 0.998, and MCC of 0.997. While the models by King et al. produced lower scores in these evaluation metrics, by comparison, they identified amino acid sequence and physiochemical feature changes by examining amino acid connectivity trends among avian and human hosts [16]. Amino acid connectivity was identified through statistical comparisons of how frequently each pairing of amino acids co-occurs. They found that while human IAVs tend to have higher mutational rates than avian IAVs, generally, the amino acid connectivity networks for avian hosts tended to be more diverse. While this is a unique approach to identify interacting features, the difference may be explained by the greater diversity of IAVs expected at this higher taxonomical level (avian) compared to a single species (human).

One study looked at the discrimination of human and swine IAV host tropism. Attaluri et al. examined the proposed swine origin of the 2009 H1N1 pandemic virus by training both SVM and decision tree classifiers to distinguish human- and swine-isolated viruses [31]. The models were tested with 2009 H1N1 pandemic sequences, the majority of which were sorted into the swine class, supporting a swine-origin of the pandemic virus.

One study looked at host discrimination beyond human, avian, and swine. Aguas and Ferguson aimed to identify markers of host-specific adaptations among five different hosts: human, avian, swine, equine, and canine [9]. PB2 amino acid sequences with conserved regions removed were converted into a matrix, and RF was used to identify patterns based on the five host labels. Metrics from this prediction model were not provided, but the authors identified 23 sequence positions as important for generating predictions.

In contrast to the previous study of multiple diverse IAV hosts, one study looked solely within the avian class. Li and Sun classified mono- and di-nucleotide composition of sequences across six species of avian hosts with a SVM [32]. The test accuracy of the model was below 0.6. The relatively poor performance of this classifier could be explained by the significant overlap in the strains commonly found in the avian species selected that created a difficult classification problem, to begin with. Five of these species from which sequences were obtained were in the genus *Anas* (ducks) which tend to host similar IAV strains [56].

### Human adaptability

A natural extension of host discrimination is predicting the zoonotic potential of non-human IAVs. This extension is exemplified in two studies that followed the aforementioned study by Eng and colleagues in 2014 [11]. After training the RF to determine features of avian or human host adaptations from amino acid physiochemical properties, they assessed presence of those identified signatures in known zoonotic strains [10]. Zoonotic avian IAV strains tended to have a mosaic of these human and animal signatures. They developed another RF to distinguish these zoonotic mosaic signatures from those of avian- or human-adapted strains [12]. Their final model had a test accuracy of 0.9714 and AUC of 0.999. Other studies looking to predict human adaptability of avian IAVs also had relative success with RF [21–24].

SVMs [20–22,24,33,34] and NNs [23,41] have also been applied with high accuracy. One unique example with NNs was reported by Qiang and Kou in 2010 [41]. The training data for this work included molecular patterns defined in a study by Kou et al. which used the unsupervised hierarchical clustering among nucleotide sequences transformed into signals [55]. The model grouped the sequences into five clusters, of which two aligned with a human-adaptable phenotype based on the 1918 pandemic IAV signal clustering. The NN developed by Qiang and Kou in 2010 discriminated between these two categories of molecular patterns [41]. They found that molecular patterns associated with human adaptable viruses were predicted with an average test error of 0.0125 and non-human adaptable viruses with 0.0092.

Another notable example comes from Wang and colleagues, who present the only study found in this review to acknowledge the inherent bias of training a model to predict human adaptability using samples where such adaptability has not consistently been assessed [35]. In doing so, instead of using a two-class human adaptable and non-human adaptable labelling scheme, Wang et al. [35] used a one-class SVM. With this method, the authors trained the model with only human-isolated avian IAVs. The model was then used to test samples for their likeness to the training data. The final model produced a test accuracy score of 0.9257. In a similar manner, Sun et al. developed a model to predict the human adaptability of avian H7N9 viruses in which the researchers intentionally biased their model to correctly classify viruses isolated from humans, such that avian viruses with high zoonotic potential would be misclassified as isolated from humans [54]. They went on to show that viruses predicted with a higher probability to infect humans grew better in mammalian cell lines and caused more severe signs of illness in mice than viruses predicted to have a lower probability of infecting humans.

### Subtype and clade assignment

While assigning the subtype of a new IAV sequence is done with alignment tools like BLAST, this method is not reliable for low homology sequencing. Therefore, some studies addressed this limitation through machine learning.

Eight studies used machine learning techniques for subtype [42,43,49,25,36,38,39] or clade assignment [37]. The most notable model by Wang et al. [35]produced separate binary predictions for each HA and neuraminidase (NA) subtype trained with amino acid matrices and combined the predictions into an ensemble model. If the input did not align with any known subtypes model would designate the sequence as a new subtype. To demonstrate this capability, the researchers temporally separated the sequences used so some newly discovered subtypes (H17, H18, N10, and N11) were only present in the test set. The model successfully identified new subtypes, and the final average test accuracy scores were 0.9943 for HA and 0.9964 for NA subtypes [43].

Shepard et al. used a SVM to predict the clade of H5 and H9 sequences [37]. Their model, which classified profile Hidden Markov Model scores of nucleotide sequences, achieved an accuracy score of 1.00 on an independent test set for both H5N1 and H9N2 clades. They further demonstrated that even partial sequences could be classified with accuracy scores between 0.88 and 0.99 if only HA1 fragments were provided. With the cleavage site removed, HA1 sequence fragments could be classified with an accuracy of 0.87 for H5N1 strains.

### Pandemic lineage assignment

Beyond subtype and clade assignments, five studies captured in this review looked at pandemic lineage assignments. Kargarfard et al. identified markers to distinguish seasonal from 2009 pandemic H1N1 [53]. Their classification based on association rule model produced a test accuracy score of 0.9960, and 10 amino acid residues were identified as potentially important determinants of class. In a subsequent report, Kargarfard et al. trained three different machine learning models with complete amino acid and nucleotide sequences for a similar objective [50]. They again found that the HA protein consistently generated the most accurate predictions with the classification based on association rules model, boasting test scores as high as 0.9999. A larger dataset was used to train and test this model, which could account for its improved performance. One other study, by Hu, used complete amino acid sequences and RF to identify 18 amino acid markers that were important for the classification of pandemic H1N1 versus other IAV sequences [13].

The remaining two studies included avian or swine IAVs in the training data and used only HA sequences for training. Meroz et al. used separately trained alternating decision trees to distinguish between (1) human H1N1 and 2009 pandemic H1N1 and (2) swine H1N1 and 2009 pandemic H1N1 [51]. For the first objective, the model produced predictions with a test accuracy of 0.98 and 10 amino acid residues were identified as important for classification. For the second objective, the model had a lower test accuracy score of 0.90, and 13 amino acid residues were found to be important for classification. Similarly, Aguas and Ferguson approached these same objectives with a RF algorithm, including a multi-class RF algorithm to classify human H1N1, human 2009 H1N1 pandemic, and swine H1N1 [9]. They directly compared their results to those of Meroz et al. [51] and found that the RF approaches produced lower prediction errors. Additionally, they identified 49 positions in the receptor binding domain (26 in known antigenic sites) as important for classification.

### Characteristics of infection

Some studies in this review looked at characteristics of IAV infection including infectivity, transmissibility, pathogenicity, virulence, or mortality. There were two studies identified in this area that have trained models with data from notable outbreaks. Allen et al. categorized viruses that have caused major epidemics or pandemics in humans, including 1918, 1957, and 1968 pandemics, human H5N1, and the 1976 H1N1, as “high mortality,” while other human IAV were considered “low mortality” [30]. The researchers then used these data to train a SVM. The model produced predictions with a test accuracy score of 0.966. Chadha et al. developed a model to classify H5 avian IAVs based on high and low pathogenicity [44]. Via a convolutional NN, the test accuracy achieved was 0.992.

Long et al. compared amino acid positions identified by RF and ADABOOST to a list of known markers of infectivity, transmissibility, and pathogenicity [28]. Of 20 known markers, 9 were identified by ADABOOST and 13 by RF as features important for determining classification. Two other studies identified in this search investigated similar characteristics but through a meta-analysis of IAV genomic patterns associated with specific disease outcomes in lab animal models. Ivan and Kwoh obtained 555 records from studies of IAV virulence in mice and assigned each sequence to a two-class (avirulent or virulent) and three-class (low, intermediate, or high) virulence category [27]. Four different machine learning algorithms were compared. PART produced the most accurate predictions across different subtypes and strains of mice for two-class test dataset scores between 0.650 and 0.844 and three-class test dataset scores between 0.540 and 0.666. Similarly, Peng et al. classified IAV sequences by virulence in mice and ferrets [29]. They obtained 228 records of studies of IAV virulence in mice and/or ferrets and used these data to train several different machine learning algorithms. The best predictions were produced by a Naïve Bayes model trained with combined nucleotide and amino acid site data resulting in a test accuracy of 0.80, sensitivity of 0.79, specificity of 0.80, and AUC of 0.85. The researchers tested the most important nucleotide positions identified by the algorithm *in vivo* through experiments in mice. Interestingly, three nucleotide sites in PB1 leading to synonymous mutations in two different IAV strains resulted in significantly lower survival rates and greater weight loss when compared to their respective wild-type strains. This not only demonstrates how powerful these predictions might be, but also underscores the importance of single nucleotide changes which the authors suggest may affect virion packaging, transcription and translation, or interactions with the host immune response.

### Antiviral drug resistance

Two studies captured in this review aimed to predict antiviral resistance via machine learning techniques [45,46]. Both numerically coded 2009 H1N1 pandemic-like nucleotide sequences, performed PCA, and finally generated predictions with decision trees and NNs. The first, by Shaltout et al. 2015, looked at resistance to the M2 ion channel inhibitor adamantane [45]. The best results were achieved with the decision tree trained with M sequences, which produced a test accuracy score of 0.982, sensitivity of 0.980, specificity of 0.986, and precision of 0.973. The second study looked at resistance to the NA inhibitor, oseltamivir [46]. In contrast to the findings of the previous study, this study found that a NN trained with NA sequences was the more successful predictor with a test accuracy score of 0.983, sensitivity of 0.980, specificity of 0.985, and precision of 0.985.

## Discussion

### Overview

In the last decade, there have been concerted efforts by researchers, national and global health organizations, as well as research funding agencies to support IAV research and create public repositories to encourage data sharing. Despite the increasing availability of sequence data, the information gained is predominantly from alignment and bioinformatics. While these tools are incredibly valuable, viral phenotype prediction likely consists of more complex relationships than can be gleaned through these techniques alone. Given this, there is increasing interest in applying machine learning algorithms to predict IAV phenotypes.

### Study aim development

The studies identified in this review predominantly developed machine learning classifiers. The main purposes of these classifiers include (1) generating predictions for new samples and (2) understanding what features are determinants of the label. Study findings and models in this area would be more widely explored and implemented if they involved interdisciplinary collaboration in their development to address questions of greater relevance to influenza biologists. Perhaps not surprisingly, the majority of studies here aimed to identify markers of host tropism and predict zoonotic potential of IAV. However, nearly all attempts to do so have relied solely on the host from which the IAV strain was isolated for assigning a classification label. Such an approach is inherently biased in that it assumes that the host from which the virus was isolated is the only possible host and ignores the possibility that certain IAVs in certain hosts have eluded surveillance efforts. One exception where there is room for improvement is the one-class SVM applied by Wang et al. [35]. This outlier identification approach alleviates some of this bias. Future studies looking to develop similar models with improved performance could explore different feature sets that may have greater predictive power and train different one-class learning algorithms, such as isolation forest (tree-based one-class classifier). Another example where this host of origin bias was accounted for was the study by Sun et al., in which the researchers intentionally biased their model to predict human labelled viruses with 100% accuracy [54]. In doing so, the model produced a probability of the human adaptability of avian H7N9 viruses which was confirmed *in vitro* and *in vivo*. Future studies could apply such an approach to H5 and other IAV subtypes where enough human isolated sequences are available.

While surveillance data labelled by host of isolation are certainly easier to obtain for training a model that assesses host tropism or zoonotic potential of viruses, it is not the most biologically sound approach. Future studies in this area would benefit from considering the host barriers that put selective pressures on IAVs and could lead to the emergence of viruses that infect hosts across different taxa. These have previously been summarized in great detail [57–59], and briefly include HA receptor binding specificity, HA stability, polymerase compatibility, NS1 interferon antagonism, antigenic distance from recently circulated viruses, and drug resistance. The comparability of these characteristics across taxa should also be considered in developing study aims, as the classification of viruses by hosts taken in many of these studies compared the avian class, suid family, and human species, which may not be appropriate. For instance, questions pertaining to receptor specificity may be more relevant at the level of class (avian versus mammalian), while innate antiviral immune responses in animals have more complex evolutionary histories. Despite the early appearance of Toll-like receptors and RIG-I-like receptors in animal evolution, they continue to undergo rapid and continued evolution with lineage-specific adaptations or losses of these receptors [60]. For example, RIG-I is absent in chickens but present in ducks [61]. Therefore, the taxonomic level of host at which these virus characteristics can be differentiated should be taken into account.

While grouping viruses by such disparate taxa may not be appropriate, cross-taxa transmission between non-human animals is underexplored with machine learning techniques. Though the study by Li and Sun was likely too granular to achieve good performance metrics, examining an ecology of wide virus promiscuity [32], similar techniques could be applied to discriminate IAV found in more taxonomically (e.g. Anseriformes versus Charadriiformes) or ecologically (e.g. wild versus domestic) distinct avian hosts. Beyond host adaptation-related questions, prediction of characteristics of infection, such as pathological effects or transmission dynamics, could be further explored with machine learning techniques.

Classification of viral genomic or proteomic data based on these cellular, immunological, and infection characteristics could be addressed through experimentally developed labels. While this approach has seen limited use in the IAV research field, successes in studies that have used machine learning techniques to identify the coreceptor usage of human immunodeficiency virus based on nucleotide or amino acid sequence data with experimentally produced labels support the efficacy of this approach [62–64]. For example, one study by Kieslich et al. used a SVM trained with nucleotide sequence data from the domain that interacts with coreceptors to predict CCR5 or CXCR4 coreceptor usage [64]. This model was found to produce predictions with an AUC of 0.977. The meta-analysis approaches utilized by Ivan and Kwoh and Peng et al. also demonstrate that there is promise in training models with experimentally generated data for IAVs [27,29]. Of course, experimental data collected in a more consistent and controlled manner would be preferred; however, the sheer number of samples needed to train a model for such complex questions could be prohibitively expensive and time-consuming for one lab to perform. With pandemic preparedness seeming more important now than ever, such models with actual predictive capability are deserving of a more concerted effort on behalf of the scientific community. Databases like IRD recognize the utility of experimental metadata, but for this to have an impact on the field, there needs to be a greater impetus on researchers to generate and report such metadata in these databases, as these reporting options are currently underutilized [4].

### Model building and reporting

Once the study question is defined, the biological level at which the question might be answered (genomic, proteomic, or physicochemical, for example) should be considered during preprocessing. The significance of this consideration is exemplified by the unexpected finding presented by Peng et al. that single, synonymous nucleotide mutations identified through machine learning led to dramatic changes in virulence in mice [29]. Given this, it may be worthwhile to train with multiple feature sets and compare their outputs to identify what level supplies the best predictive power for classification. The predictive power of the sample data also needs to be preserved through transformations into input features and any potential dimensionality reduction that is performed. At these early model development stages, efforts should be made to create models that are generalizable enough to phenotypically characterize IAV strains collected across different virus subtypes. If it is necessary to separate by subtype to achieve the desired model performance, combining binary subtype specific classifiers into an ensemble, as was done in the earlier described subtype assignment model developed by Wang et al. [43], may be preferable.

### Study follow-up

**Figure 4.**
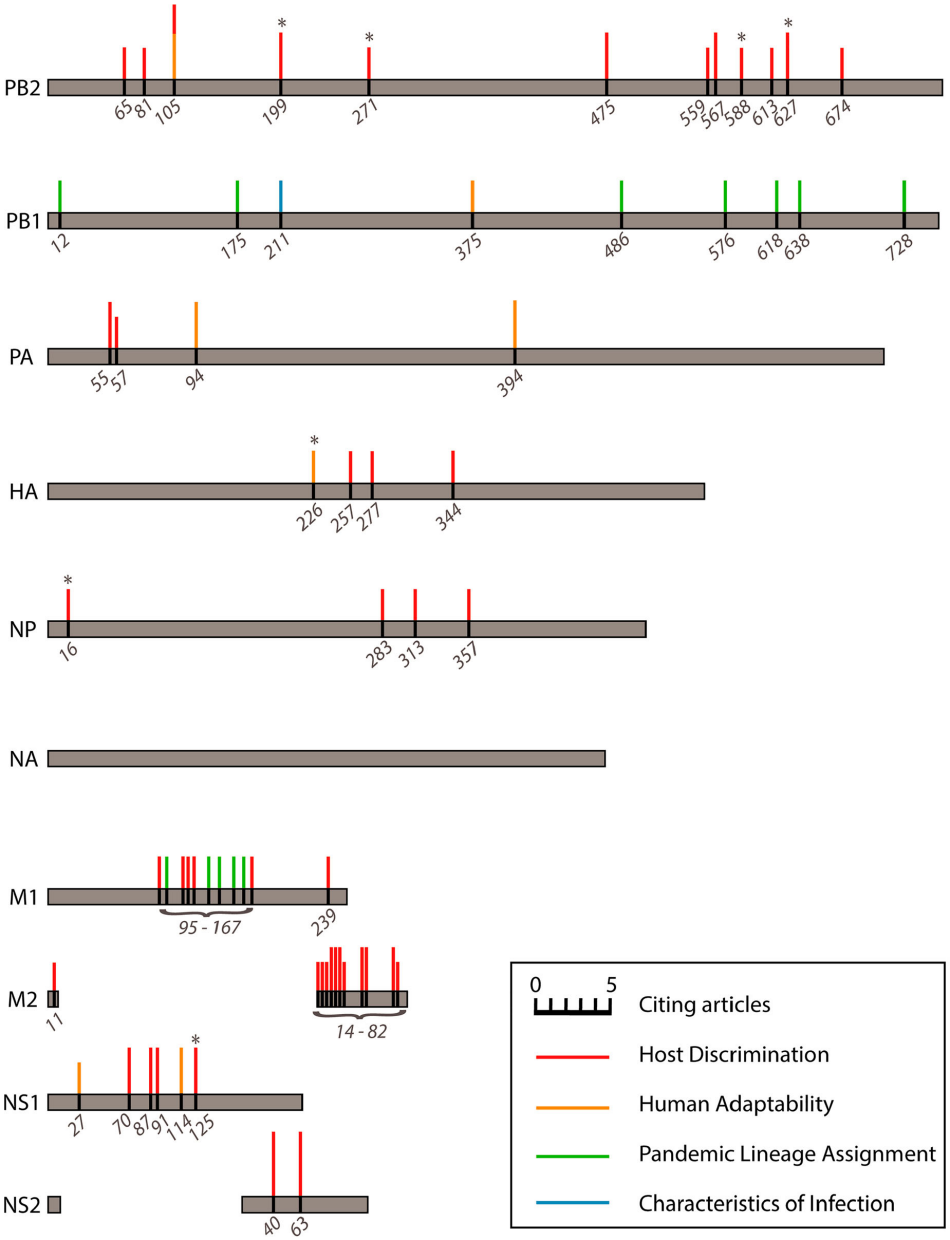
Influenza A virus amino acid positions identified as important for generating predictions of host discrimination, human adaptability, pandemic lineage assignment, or characteristics of infection among two or more of the reviewed machine learning studies from independent labs. The size of the coloured bars is proportional to the number of references citing that position as an important feature in their model. No important features in neuraminidase (NA) were identified in more than one study. Asterisks denote positions with an empirically demonstrated function; see Table S3 for descriptions and references.

In addition to developing experimentally derived training data, there is also a need for more extensive experimental follow-up studies. Peng et al. and Sun et al. were the only studies captured in this review to test their computational predictions *in vitro* and/or *in vivo* [29,54]; though it should be noted that similar follow-up studies may be separately published and were, thus, not captured in the literature search, though none were found during citation tracing. The follow-up performed in these two studies looked at features predicted to contribute to label assignment in their respective models. Algorithms such as RFs and SVMs have “built-in” methods for determining feature importance. For these and other algorithms, there are a number of other methods for investigating feature importance such as through permutation of specific features [65], iterative optimization of random inputs to elicit hidden layer excitement in NNs [66], or systematic removal of each feature during model training to assess its impact on model performance. Specific amino acid substitutions identified as important features in two or more studies included in this review are summarized in Figure 4 and Table S3. It should be noted that the selection of specific amino acid substitutions presented in Figure 4 and Table S3 was dependent on the discretion of the authors of their respective reports presumably based on thresholds deemed appropriate given their model parameters or outcomes, as well as the specification of the HA numbering scheme used to present the positions. While post experimentation of such amino acid changes for functional relevance may be limited in some instances if it encroaches on a gain of function research, other ways to test predictions on the bench, such as through pseudotype viruses with questions concerning membrane-bound proteins [67], could be explored to appropriately establish function [68].

### Review limitations and conclusions

While the aim of this review is to be comprehensive, it is possible that some relevant studies were not captured in this search. It is also important to note that while this review sought to compare studies with similar aims directly, the training and testing sets across these studies are not consistent, which can impact the success of a model. Nonetheless, many studies captured in this review demonstrate that there is great potential to produce accurate predictions of IAV phenotypic traits with sequence data using machine learning approaches and contribute to the repertoire of known genomic or proteomic contributors to phenotypic traits. These results, therefore, call for attention from influenza biologists and computer scientists to continue to develop improved models that could be used as part of a pandemic preparedness plan.

## Supplementary Material

Machine_Learning_and_Influenza_Review_Supporting_Info_-_clean.docx
