# Supplementary material for: Influenza virus genotype to phenotype predictions through machine learning: a systematic review: Computational Prediction of Influenza Phenotype
Source: Emerg Microbes Infect. 2021 Sep 23;10(1):1896–907. doi: 10.1080/22221751.2021.1978824 (PMC8462836; doi:10.1080/22221751.2021.1978824)
Supplement: Machine_Learning_and_Influenza_Review_Supporting_Info_-_clean.docx [file TEMI_A_1978824_SM6415.docx]

**Supplemental Information**

| **Host Discrimination** | | | | | |
| --- | --- | --- | --- | --- | --- |
| **Publication** | **Objective(s)** | **Data Used** | **Data Processing** | **Technique(s) Used** | **Model Performance Metrics** |
| [1] Aguas and Ferguson 2013  (cross-listed) | To identify markers of host specific adaption in the amino acid sequence of PB2 genes from multiple hosts and subtypes of IAV. | 543 full PB2 amino acid sequences of influenza viruses encompassing 7 influenza subtypes (H1N1, H1N2, H2N2, H3N2, H5N1, H3N8, H7N7) collected from 5 different hosts (humans, birds, pigs, dogs, and horses). | Conserved regions identified through multiple sequence alignment were removed, and a binary amino acid frequency matrix was constructed | Random Forest | Not specified |
| [2] Allen *et al*. 2009^†^  (cross-listed) | To predict host tropism of human and avian IAV. | Amino acid sequences of 2,026 human and 1,018 avian derived influenza viruses were obtained from NCBI. | Amino acid sequence | Linear Support Vector Machine | The host classification accuracy was 0.995. |
| [3] Attaluri *et al.* 2009^†^ | To further examine the proposed swine origin of the 2009 pandemic H1N1. | 150 sequences for each class of human only, swine only, and human/swine 2009 pandemic H1N1 influenza virus strains were obtained from GISAID and NCBI. | Nucleotide sequences were used for decision tree and protein sequences were used for support vector machine classifiers. | Decision Tree and Support Vector Machine | Support vector machine accuracy was consistently above 0.95 for all gene segments, with sensitivity and specificity also consistently above 0.96 and 0.97, respectively. Decision trees had accuracy above 0.96, sensitivity above 0.95, and specificity above 0.98 consistently. |
| [4] Attaluri *et al*. 2010^†^  (cross-listed) | To predict host tropism of human, avian, and swine IAV. | For human, avian and swine hosts, 100 complete genome sequences of subtypes H1, H3, or H5 were obtained from NCBI. | Tested three different nucleotide numerical coding schemes. | Neural Network | Classification accuracy across various different preprocessing schemes, different model parameters, and sequences from individual gene segments typically resulted in accuracies above 0.90 for host prediction. |
| [5] ElHefnawi and Sherif 2014^†^ | To predict host tropism of human, avian, and swine IAV. | 1500 H1, H2, H3, H5, and H9 amino acid sequences of human, swine, and avian IAV were obtained from NCBI. | VESPA and Sample Logo were used to select sequence features to include in classification. | Decision Tree and Classification Based on Association Rules | HA subtype-specific trained decision trees produced accuracies between 0.912 and 1.000. |
| [6] Eng *et al.* 2014 | To predict host tropism of human and avian IAV. | Complete sequences obtained from IRD. The training set included 20,923 human and 30,548 avian sequences, and the test set included 3,272 human and 3,923 avian sequences. | Generated 146 feature vectors based on amino acid composition, transition between property groups (hydrophobicity, normalized van der Waals volume, polarity, and polarizability), and distribution. | Random Forest selected after use of WEKA platform to identify best suited algorithm. | The model trained with vectors for all 11 proteins produced predictions with accuracy of 0.9983, sensitivity of 0.998, specificity of 1.000, AUC of 0.998, and MCC of 0.997 for the independent test set. |
| [7] Hu 2011^†^ | To predict host tropism of human, avian, and swine H5N1. | Full length amino acid sequences were obtained from NCBI. | Amino acid sequence and feature selection by random forest | Random Forest | Not specified |
| [8] Hu 2010^†^ | To predict host tropism of human, avian, swine, and pandemic H1N1. | Amino acid and nucleotide sequences of IAV were obtained from NCBI. | Nucleotide and amino acid sequences were used along with feature selection by random forest | Random Forest | Not specified |
| [9] Hu 2010^†^ | To predict host tropism of human, avian, swine, and pandemic H1N1. | Avian, human, and swine influenza viruses, including the 2009 pandemic H1N1, nucleotide sequences were obtained from NCBI. | Nucleotide sequence and feature selection by random forest | Random Forest | Not specified |
| [10]  Kargarfard *et al*. 2016 | To predict host tropism of human, avian, and swine IAV. | A total of 674 amino acid sequences were obtained from IRD, NCBI, and GISAID. | Features consisted of amino acid present at each position | Decision Trees, RIPPER, and Classification Based on Association Rules | Not specified |
| [11] Kincaid 2018^†^ | To predict IAV host | 730,106 amino acid sequences were obtained from NCBI. Report did not specify which hosts were included. | Sequences were transformed into N-grams. | Random Forest, Naïve Bayes, Linear Support Vector Machines, and Neural Network | Highest accuracy score was achieved by the neural network at 0.972. Naïve Bayes had the poorest performance with an accuracy score of 0.548. |
| [12] King *et al.* 2010^†^ | To predict host tropism of human and avian IAV. | Full length amino acid sequences were obtained from NCBI. | 131 features were selected using seven algorithms from WEKA (Chi-Squared, Information Gain, Information Gain Ratio, 1R, Relief, Symmetrical Uncertainty, and Filtered Attribute Evaluation). | Support Vector Machine, Naïve Bayes, Random Forest, and Rotation Forest. | Average scores of the four different models across all segments was above 0.92 for MCC, 0.97 for specificity, and 0.91 for sensitivity. |
| [13] Kwon *et al*. 2020^†^ | To predict host tropism of human, avian, and swine IAV. | 109,451 amino acid sequences of 6 proteins were obtained. | 146 physicochemical amino acid features were generated to represent each sequence. Random Forest feature selection was used to generate several subsets of features based on importance for classification | Random Forest, Naïve Bayes, K-Nearest Neighbors | Random forest had the best performance across the board in terms of accuracy and AUC (0.965 or higher for all proteins). |
| [14] Li and Sun 2018 | To predict host tropism of avian IAV among six avian species. | 200 sequences each for 6 avian species obtained from IRD. | Mono and dinucleotides | K-Nearest Neighbors and Support Vector Machine | Prediction accuracy among all models was below 0.6. |
| [15] Shaltout *et al.* 2015^†^ | To predict host tropism of human, avian, and swine IAV. | Nucleotide sequences of subtype H1 were obtained. | Information gain was used for features selection of the 100 most informative features per segment. | Decision Tree and Neural Network | Accuracy for both classifiers with and without feature selection performed was above 0.98. |
| [16] Sherif *et al*. 2011^†^ | To predict host tropism of human, avian, and swine IAV. | 1500 HA amino acid sequences (500/host) were obtained from NCBI IVR. | VESPA and Two Sample Logo were used to select sequence features to include in classification. | Classification Based on Association Rules | Support and confidence, respectively, of three amino acid positions identified as potential markers of host tropism were 90.7% and 79.5%, 82.8% and 92.9%, and 51.4% and 98%. |
| [17] Xu *et al.* 2017^†^ | To predict host tropism of human, avian, and swine IAV. | 163,666 unique DNA and 150,947 unique amino acid sequences were obtained from UniProt. | Word2vec was used to transform sequences. Words were of length 2-4, and they were summed and averaged s into 200-dimensional data. | Support Vector Machine | Overall accuracy ranged from 0.79-0.96 depending on the protein input. For each host (avian, human, swine) accuracy ranged between 0.97 and 1.00. |
| [18] Yin *et al.* 2018 | To predict host tropism of human, avian, and swine IAV. | 8,419 avian strains, 12,759 human strains and 5,987 swine HA (1,2,3,5,7,9) sequences were obtained from NCBI. | Amino acids were transformed into physiochemical factors (polarity, polarizability, net charge, normalized van der Waals volume, hydrophobicity, secondary structure and solvent accessibility). SMOTE was used to balance H5 and H9 oversampling. Random forest was used to separately evaluate feature influence of sequences obtained from different geographic locations. | K-Nearest Neighbor, Logistic Regression, Neural Network, Support Vector Machine, Random Forest | Random forest model trained with combined HA subtypes with SMOTE data resampling had accuracy of 0.948, precision of 0.954, and MCC of 0.922. |
| [19] Yin *et al.* 2020 | To estimate reassortment probabilities through host tropism prediction of influenza viruses. | Amino acid sequences of 13598 avian, 20614 human, and 4380 swine influenza viruses for host tropism discrimination were obtained from NCBI. Amino acid sequences from 208 known reassortant strains were obtained from GISAID. | 147 features were generated from seven physicochemical properties (polarity, net charge, hydrophobicity, normalized van der Waals volume, solvent accessibility, polarizability, secondary structure). | Random Forest | Host predictions among each viral protein showed an accuracy range of 0.865 to 0.965 on independent test sets. |
| **Human Adaptability** | | | | | |
| **Publication** | **Objective(s)** | **Data Used** | **Data Processing** | **Technique(s) Used** | **Model Performance Metrics** |
| [20] Eng *et al*. 2016 | To see if host tropism signatures identified via machine learning techniques are present in zoonotic influenza strains. | Complete sequences obtained from IRD. Training set included 12,624 human and avian amino acid sequences. Test set includes sequences from 126 confirmed and 346 suspected zoonotic strains. | Generated 146 feature vectors of amino acid composition, transition between property groups (hydrophobicity, normalized van der Waals volume, polarity, and polarizability), and distribution. | Random Forest | Based on random forest model built for Eng et al. 2014; prediction accuracy was between 0.9657 and 0.9862. |
| [21] Eng *et al.* 2017 | To use previously identified zoonotic influenza amino acid signatures to develop a computational model for predicting zoonotic influenza strains. | Complete sequences obtained from IRD. Training set included 125 avian, 127 human, and 125 zoonotic IAV strains. Test set included 34 avian, 37 human, and 35 zoonotic IAV strains. | Generated 146 feature vectors of amino acid composition, transition between property groups (hydrophobicity, normalized van der Waals volume, polarity, and polarizability), and distribution. | Random Forest selected after use of WEKA platform to identify best suited algorithm | Overall accuracy was 0.9906, weighted AUC was 1.000, zoonotic accuracy was 0.9714, zoonotic sensitivity was 0.971, zoonotic specificity was 1.000, and zoonotic AUC was 0.999 with an independent test set. |
| [22] Kou *et al.* 2008^†^ | To identify signal patterns of influenza virus sequences associated with avian to human transmission potential. | 237 IAV nucleotide sequences were obtained from NCBI and NIH. | Sequences were transformed into signals, and feature extraction by wavelet packet decomposition was performed. | Hierarchical Clustering | N/A |
| [23] Li *et al.* 2019 | To predict human adaptability of swine and avian influenza viruses. | 46,042 human-adaptive, 46,488 non-human adaptive avian, and 35, 569 swine influenza sequences were extracted from IRD and GISAID. | Used a resampling method to select random sequences from the US to avoid sample region bias. Features consisted of 60 types of mononucleotide and dinucleotide composition. PCA and support vector classifier were used for feature selection. | Gradient-Boosted Regression Trees, Neural Network, Random Forest, and Support Vector Machines | A support vector machine classifier trained on top 12 features from dinucleotide composition of PB2 sequences produced a true-negative rate of 0.9845, true positive rate of 0.9410, and AUC of 0.995. |
| [24] Qiang *et al*. 2018 | To predict human adaptability of avian influenza viruses. | 429 human-origin and 440 avian-origin avian influenza viruses were obtained from GISAID. | Avian-origin sequences with high similarity (97% or more) to human-origin were filtered out of the dataset. Each amino acid position was assigned scores for five physiochemical amino acid properties (polarity, secondary structure, molecular volume, codon diversity, and electrostatic charge) and those scores were considered both separately and as a sum with each of the different classifiers. Random Forest feature selection was performed. | Random Forest, K-Nearest Neighbors, Naïve Bayes, Support Vector Machine | Support vector machine and random forest classification had the highest average AUC near 1.0, followed shortly-thereafter by Naïve Bayes. K-Nearest Neighbors had a relatively poor AUC at 0.5. |
| [25] Qiang and Kou 2019 | To predict human adaptability of avian influenza viruses. | 429 human-origin and 440 avian-origin avian influenza viruses were obtained from GISAID. | Avian-origin sequences with high similarity (97% or more) to human-origin were filtered out of the dataset. Each amino acid position was assigned scores for five physiochemical amino acid properties (polarity, secondary structure, molecular volume, codon diversity, and electrostatic charge) and separately tested each of those scores with the different classification techniques. Wavelet packet decomposition was used for feature extraction. | K-Nearest Neighbors, Naïve Bayes, Support Vector Machine | Support vector machine had the best performance with an AUC approaching 1. The AUC was only slightly lower for Naïve Bayes. K-nearest neighbors had the poorest performance with an AUC of about 0.5. |
| [26] Qiang and Kou 2010^†^ | To predict human adaptability of avian influenza viruses. | Human-adaptable and non-human-adaptable molecular patterns were defined based on work by Kou et al. 2008. 70 amino acid sequences of the 6 internal proteins were obtained for each of those two categories. | Feature selection was performed with wavelet packet decomposition and PCA, for a final 21 features. | Neural Network | Average errors of 0.0125 and 0.0092 were observed for human-adaptable and non-human-adaptable sequences, respectively. |
| [27] Qiang and Kou 2019 | To predict human adaptability of avian influenza viruses. | 869 AIV strains (440 avian-origin and 429 human-origin) and 914 seasonal, pandemic human, and artificial strains (6 sequences obtained from separate references) were mainly obtained from GISAID. | Random forest algorithm was used to assign a score to amino acids at each position based on crude importance for transmissibility to humans. | Random Forest, Support Vector Machine, Naïve Bayes, K-Nearest Neighbors | Support vector machine and random forest had the best performance with an AUC approaching 1 and K-Nearest Neighbors was the worst with an AUC of about 0.5. |
| [28] Scarafoni *et al.* 2019 | To predict human adaptability of avian influenza viruses. | Total of 306,659 sequences spread among 7 different proteins from NCBI. | Amino acid | K-Nearest Neighbors, Random Forest, Convolutional Neural Network | Mean accuracy for test set across the 7 proteins for K-nearest neighbors was 0.979, random forest was 0.990, and a tailored convolutional neural network was 0.990. |
| [29] Sun *et al.* 2020 | To predict human adaptability of avian influenza viruses. | A total of 877 avian and human H7N9 nucleotide sequences were obtained from IRD. | Nucleic acids were coded as integers. | Class Weight Biased Logistic Regression | Model was intentionally biased to predict human labels with 100% accuracy. |
| [30] Wang *et al.* 2013 | To predict human adaptability of avian influenza viruses. | Data obtained from IRD and NCBI. This consisted of amino acid sequences from 6 inner proteins of 98 human-isolated and 1784 avian-isolated avian influenza viruses. | Score of 5 physiochemical amino acid factors (polarity, secondary structure, molecular volume, codon diversity, and electrostatic charge). 87 amino acid positions were extracted based on differences in information entropy at each position for human and avian hosts, with a cutoff of 0.5. | Decision Tree, Naive Bayes, Random Forest, Support Vector Machine | AUC medians are higher than 0.95 across all classifiers. Naïve Bayes produced the score optimum between train and test folds. |
| [31] Wang *et al.* 2013 | To predict human adaptability of avian influenza viruses. | Data obtained from IRD and NCBI. This consisted of amino acid sequences from 6 inner proteins of 78 human-isolated and 1173 avian-isolated avian influenza viruses. | 90 amino acid positions were extracted based on differences in information entropy at each position for human and avian hosts, with a cutoff of 0.5. Each of these sequences was converted into 531 physiochemical features. Four different feature selection methods were applied to choose the 40 most informative features. | Support Vector Machine | The model produced a sensitivity of 0.8889, specificity of 1.00, MCC of 0.939, accuracy of 0.9931 for the testing data. |
| [32] Wang *et al.* 2012^†^ | To predict human adaptability of avian influenza viruses. | 78 human- and 175 avian-isolated avian influenza virus sequences were obtained from NCBI IVR and IRD. 60 human-isolated strains were used for training. | Sequences were converted into the following four different features sets: amino acid composition, dipeptide composition, autocorrelation feature, and performance evaluation. | One-Class Support Vector Machine | Best prediction accuracy was produced with amino acid composition and autocorrelation feature training dataset with a score of 0.9257. |
| **Subtype Assignment** | | | | | |
| **Publication** | **Objective(s)** | **Data Used** | **Data Processing** | **Technique(s) Used** | **Model Performance Metrics** |
| [33] Attaluri *et al.* 2009^†^ | To predict IAV subtype | 2,154 HA (1-3) and 2,259 NA (1-2) sequences were obtained from NCBI. | Nucleotide sequence | Decision Tree | Preliminary testing produced accuracy of 1.00 (data not shown). |
| [4] Attaluri *et al*. 2010^†^  (cross-listed) | To predict HA subtype | For human, avian and swine hosts, 100 complete genome sequences of subtypes H1, H3, or H5 were obtained from NCBI. | Tested three different nucleotide numerical coding schemes. | Neural Network | Classification metrics for their Type 2 processing scheme demonstrated an accuracy of 0.92, sensitivity of 0.93, and specificity of 0.91. For their Type 3 processing scheme accuracy was 0.92, sensitivity was 0.92, and accuracy was 0.93. |
| [34] Chrysostomou and Seker 2013 | To predict NA subtype | NA amino acid sequences of 200 H1N1 from 2009, 76 H2N2 from 1957-1968, 200 H3N2 from 1968-200, and 70 H5N1 from 2005-2009 were collected from NCBI. | Amino acid sequences were transformed into EIIP numerical codes and DFT-based features were extracted. F-score statistical feature selection was performed. | Support Vector Machine | The overall accuracy of the model was 0.983, and it ranged between 0.920-1.00 for each specific subtype. |
| [35] Ebrahimi *et al.* 2014 | To predict HA subtype | 7338 HA records of subtypes H1 to H16 | 868 amino acid physicochemical attributes were calculated for each HA sequence. Ten different algorithms for feature weighting were tested. | Decision Tree, Support Vector Machine, Naïve Bayes, Neural Networks, Random Forest | The random forest model with Gini Index weighted features had accuracy of 0.9970. Neural networks with a variety of filtering and weighting also produced accuracy scores in the 0.997 range. |
| [36] Fabijańska *et al.* 2019^†^ | To predict IAV subtype | 2 different subsets of influenza virus nucleotide sequences were obtained from NCBI containing 313,782 and 317,728 genomes. | Nucleotides were converted into ASCII codes. | Convolutional Neural Network | Their best influenza virus model produced test metrics on the 2nd dataset with accuracy of 0.978, sensitivity of 0.841, specificity of 0.982, precision of 0.835, and F1 of 0.835; scores were comparable for the 1st test dataset. |
| [37] Humayun *et al*. 2021 | To predict IAV subtype | 26,586 HA (1-16) and 20,690 NA (1-9) nucleotide sequences were obtained from NCBI. | Physicochemical properties were derived from the nucleotide sequence and feature extraction was performed. | Decision Tree, Support Vector Machine, Naïve Bayes, K-Nearest Neighbor | The highest observed accuracy score of 0.9514 was achieved with the decision tree. |
| [38] Shepard *et al.* 2014 | To predict H5 and H9 clade | 645 H5N1 and 342 H9N2 hemagglutinin nucleotide sequences were obtained from NCBI, GISAID, and WHO. | Profile hidden Markov model scores of nucleotide sequences were generated. | Support Vector Machine | Accuracy score of 1.00 was achieved for both full length H5N1 and H9N2. Partial sequences showed accuracy of 0.87-0.99. |
| [39] Wang *et al.* 2020^†^ | To predict IAV subtype | 59,581 HA and 40,072 NA sequences from 2010 onward were obtained from NCBI. Training and test sets were separated temporally to demonstrate potential to generate predictions for subtyping of future strains. | Amino acid sequences are converted into a matrix. | Convolutional Neural Network | Overall accuracy of HA subtyping was 0.9943 with F1 of 0.9943. Accuracy of NA subtyping was 0.9964 with F1 of 0.9964. |
| **Pandemic Lineage Assignment** | | | | | |
| **Publication** | **Objective(s)** | **Data Used** | **Data Processing** | **Technique(s) Used** | **Model Performance Metrics** |
| [1] Aguas and Ferguson 2013 (cross-listed) | To classify swine, human, and pandemic H1N1 HA amino acid sequences. | 866 pre-pandemic H1N1, 674 pandemic human H1N1 (pH1N1), and 368 swine H1N1 full HA segment amino acid sequences. | Conserved regions identified through multiple sequence alignment were removed, and a binary amino acid frequency matrix was constructed | Random Forest | The multi-class random forest algorithm (human vs human pandemic vs swine) produced a prediction error of 0.016. |
| [40] Hu 2010^†^ | To identify amino acid markers to distinguish 2009 pandemic H1N1 from non-pandemic H1N1. | Avian, human, and swine influenza viruses, including the 2009 pandemic H1N1, amino acid sequences were obtained from NCBI. | Amino acid sequence with feature selection by random forest | Random Forest | Not specified |
| [41] Kargarfard *et al*. 2015^†^ | To classify 2009 pandemic H1N1 and non-pandemic H1N1 HA sequences | 3621 pandemic and 1752 non-pandemic HA sequences were obtained from IRD. | Nucleotide and amino acid sequences were used | Classification Based on Association Rules | With an independent test data set, the model produced an accuracy score of 0.9960, sensitivity of 1.0000, specificity of 0.9245, and precision of 0.9958. |
| [42] Kargarfard *et al.* 2019 | To classify 2009 pandemic H1N1 and non-pandemic H1N1 HA sequences | Between 4226 and 5373 sequences of 10 different influenza virus proteins of 2009 H1N1 pandemic and seasonal sequences were extracted from IRD. | Features consisted of amino acid or nucleotide present at each position | Decision Trees, RIPPER, and Classification Based on Association Rules | Highest classification accuracies were observed with a classification based on association rules model trained with HA sequences; HA nucleotide sequences were classified with 0.9999 accuracy. |
| [43] Meroz *et al*. 2011^†^ | To classify 1) human seasonal H1N1 versus 2009 pandemic H1N1 HA sequences and 2) swine H1N1 versus 2009 pandemic H1N1 HA sequences. | The dataset for the first objective consisted of HA sequences of 706 2009 pandemic H1N1 and 852 prior circulating human strains, excluding the 1918 pandemic strain. The dataset for the second objective consisted of 245 swine H1N1 and 782 2009 pandemic H1N1 HA sequences. Sequences were obtained from NCBI. | Amino acid sequence | Alternating Decision Trees | Accuracy for human seasonal H1N1 versus 2009 pandemic H1N1 was 0.98 and swine H1N1 versus 2009 pandemic H1N1 was 0.90. |
| **Characteristics of Infection** | | | | | |
| **Publication** | **Objective(s)** | **Data Used** | **Data Processing** | **Technique(s) Used** | **Model Performance Metrics** |
| [2] Allen *et al*. 2009^†^  (cross-listed) | To identify markers in strains that lead to high and low mortality rates. | Amino acid sequences of 2026 human and 1018 avian derived influenza viruses were obtained from NCBI. High mortality strains included sequences from the 1918, 1957, and 1968 outbreaks, human H5N1, and the 1976 H1N1 that lead to deadly outbreaks in New Jersey. All other human influenza viruses were classified as low mortality. | Amino acid sequence | Linear Support Vector Machine | Average accuracy of mortality classifications was 0.966. |
| [44] Chadha *et al*. 2019^†^ | To classify H5 avian influenza virus strains based on pathogenicity. | 1202 highly pathogenic and 1167 low pathogenic H5 avian influenza sequences were obtained from IRD, NCBI, and previous studies. | Amino acid sequences were numerically coded where each amino acid position was represented by a numerical feature-specific to each possible amino acid. | Convolutional Neural Network | Mean accuracy was 0.992. |
| [45] Ivan and Kwoh 2019 | To identify genetic predictors of influenza virus virulence in mice. | 555 records were obtained from studies of IAV virulence in mice through a literature search. The associated sequences were obtained from NCBI and GISAID. | Amino acid sequences were used. Rules were designed to assign ordinal virulence two- (avirulent and virulent) and three-class (low, intermediate, high) outcomes. | OneR, JRip, PART, and Random Forest | Average accuracy over models for two-class datasets was 0.650-0.844 and for three-class dataset was 0.540-0.666. |
| [46] Long *et al.* 2019 | To compare machine learning identified genetic predictors of the infectivity, transmissibility, and pathogenicity to positions with empirically demonstrated function. | Full amino acid sequences were obtained from NCBI for 10 strains. | Binary values for infectivity, transmissibility, and pathogenicity were assigned by strain based on IRD phenotype data. | ADABOOST and Repeated Random Forest | Not specified |
| [47] Peng *et al.* 2020^†^ | To identify genetic predictors of influenza virus virulence in mammals (ferrets and mice). | 228 records were obtained from studies of IAV virulence in mice and/or ferrets through a literature search performed on PubMed. The associated sequences were obtained from NCBI and GISAID. | Nucleotide and amino acid sequences were used. Fisher's exact test (p-value) and q-value were used to select features with significant association (p<0.05 and q<0.05) with high virulence. 111 nucleotide positions were used for final ML models. | Decision Tree, Random Forest, Logistic Regression, Neural Network, and Naïve Bayes | The best predictions were produced by a Naïve Bayes model trained with combined nucleotide and amino acid site data resulting in an accuracy score of 0.80, sensitivity of 0.79, specificity of 0.80, and AUC of 0.85. |
| **Drug Resistance** | | | | | |
| **Publication** | **Objective(s)** | **Data Used** | **Data Processing** | **Technique(s) Used** | **Model Performance Metrics** |
| [48] Shaltout *et al.* 2015^†^ | To predict adamantane resistance | Matrix nucleotide sequences resembling 2009 pandemic H1N1 from human, avian, or swine hosts with and without adamantane resistance noted were obtained from IRD. | Sequences were converted to numerical codes and PCA was used for feature selection. | Decision Tree and Neural Network | Decision tree produced an accuracy score of 0.982, sensitivity of 0.980, specificity of 0.986, and precision of 0.973. |
| [49] Shaltout *et al.* 2016^†^ | To predict oseltamivir resistance | NA nucleotide sequences resembling 2009 pandemic H1N1 from human hosts with and without oseltamivir resistance noted were obtained from IRD. | Sequences were converted to numerical codes and PCA was used for feature selection. | Decision Tree and Neural Network | The best predictions were produced by the neural network trained with 40 PCA features with an accuracy score of 0.983, sensitivity of 0.980, specificity of 0.985, and precision of 0.985. |

| **Table S2. Descriptions of the most frequently used feature selection, machine learning, and evaluation techniques used to generate influenza virus genotype to phenotype predictions.** | | | |
| --- | --- | --- | --- |
| **Feature Selection** | | | |
| **Technique** | | **Brief Description** | **Advantages and Disadvantages** |
| Information Gain | | Information gain is a measure of how much entropy (unpredictability) is removed from a dataset and can be used to set threshold for feature selection. | This and other filter feature selection methods are useful for determining correlation of features with the class label but overlook features with a synergistic effect that may contribute to class label assignment. |
| Principle Component Analysis | | Feature extraction technique that transforms features onto a new, lower dimensional, coordinate plane that explains the amount of variability in the data based on the correlation of features between samples | This and other feature extraction techniques can drastically reduce dimensionality by fitting features to a new coordinate plane; however, this transformation is irreversible and can, therefore, make interpretation of individual feature importance difficult. |
| Random Forest | | Ensemble of decision trees generated by bootstrap aggregation of the training set from which feature importance can be derived based on heuristics such as information gain or Gini impurity. A feature selection cutoff can then be chosen either by setting an importance threshold or choosing *n* most important features all at once or through recursive application of the algorithm for tree construction, providing for selection that is more flexible than with information gain by itself. | Embedded feature selection techniques, which exist within a machine learning algorithm such as random forest, tend to be relatively more accurate and generalizable than other feature selection techniques. However, tree-based techniques may need to be run recursively to identify correlated features that have a synergistic relationship with the class label, and they tend to be biased toward features with high cardinality (more distinct values). |
| **Machine Learning Algorithms** | | | |
| **Algorithm** | | **Brief Description** | **Advantages and Disadvantages** |
| ADABOOST | | Sequentially trains weak classifiers, like decision stumps (one-level decision trees), each learning from the weaknesses of the last, and combines them to perform as an ensemble. | It is a simple classifier, but it is not well suited to high dimensionality data, is susceptible to overfitting, and is not useful for continuous data. |
| Classification Based on Association Rules | | Predicts class label based on the presence of sets of associated features. | It is a simple approach to classification, but it is susceptible to overfitting. |
| Decision Trees | | Paths of sorted feature values terminating in discrete classes. | They are intuitive and requires less preprocessing of data (no normalization, scaling, or imputing missing values is needed), but they can be time consuming to train and are harder to apply to continuous feature values. |
| K-Nearest Neighbors | | Groups a specified number of samples (K) with class-labelled points by distance across an n-dimensional feature space. | It is a simple algorithm that works with multi-class problems. However, it is not well suited for high dimensionality data and is very sensitive to outliers. Data normalization can improve performance. |
| Logistic Regression | | Uses maximum likelihood to fit a sigmoid function to binary classified data, generating a probability of the class label. | It is simple to implement but may require dimensionality reduction, because it does not work well with large feature spaces, high dimensional data, and can perform poorly when there are many extraneous and uninformative features or when many features are strongly correlated. |
| Naïve Bayes | | Assigns the most likely class label based on the probabilities of specific combinations of features presented in the training data, with the assumption that all features are independent of each other. | It is simple to implement and works well with high dimensional data. However, features with a probability of zero will be unable to be classified without correction. It is also not useful for continuous feature values, and the assumption of independence may be problematic for certain classification problems. |
| Neural Network | | Uses layers of hidden algorithms to predict class labels from input features. | They can be tuned to produce highly accurate predictions from complex, high dimensional data; however, they are a “black box” in that it is difficult to identify features important for generating predictions. They may also require very large datasets to outperform or even match what can be done with simpler regression or tree-based methods. |
| Random Forest | | Ensemble of decision trees generated by bootstrap aggregation of the training set. | Works well with high dimensional data, is less prone to overfitting, and it is relatively easy to derive feature importance. It is computationally time consuming to train. |
| Support Vector Machine | | Identifies a hyperplane that separates the nearest datapoints of different classes, transforming the data with a kernel function to create this separability where necessary. | Works well with high dimensional data and is less susceptible to outliers. However, it is slow to implement, and it requires a lot of hyperparameter tuning. |
| **Performance Evaluation** | | | |
| **Technique/Metric** | **Definition** | | |
| Accuracy | Proportion of correct predictions out of all generated predictions. | | |
| AUC | Area under the curve of true positive rate plotted against false positive rate. | | |
| Cross-validation | The separation of data into multiple folds to use for training and testing to prevent overfitting during model development. | | |
| F1 Score | Harmonic mean of precision and sensitivity. | | |
| MCC | A correlation coefficient ranging between -1 and 1, where -1 is an incorrect prediction and 1 is a correct prediction. | | |
| Precision | Proportion of true positive predictions out of all predicted positives. | | |
| Sensitivity | Proportion of true positive predictions out of all actual positives. | | |
| Specificity | Proportion of true negative predictions out of all actual negatives. | | |
| AUC = area under the receiver operating characteristic curve  MCC = Matthew’s correlation coefficient | | | |

| **Table S3. Influenza A virus amino acid positions identified as important for generating predictions of host discrimination, human adaptability, pandemic lineage assignment, or characteristics of infection among two or more of the reviewed machine learning studies from independent labs.** | | |
| --- | --- | --- |
| **Amino acid position** | **Identifying references** | **Relevant empirically demonstrated function** |
| PB2 65 | Host discrimination [1, 9] | None found |
| PB2 81 | Host discrimination [1, 9] | None found |
| PB2 105 | Human adaptability [27, 30, 31] and host discrimination [1, 9] | None found |
| PB2 199 | Host discrimination [1, 2, 9] | H5N1 PB2 A199S increased virulence in mice [50] |
| PB2 271 | Host discrimination [1, 9] | PB2 T271A increased polymerase activity in avian and mammalian cell lines for H3N2 [51], H5N1 [52], and H7N9 [53] |
| PB2 475 | Host discrimination [1, 2, 9] | None found |
| PB2 559 | Host discrimination [1, 9] | None found |
| PB2 567 | Host discrimination [1, 2, 9] | None found |
| PB2 588 | Host discrimination [1, 9] | PB2 A588V increased polymerase activity and replication in mammalian and avian cell lines, as well as increased virulence in mice for H7N9, H9N2, and H10N8 [54] |
| PB2 613 | Host discrimination [1, 9] | None found |
| PB2 627 | Host discrimination [1, 2, 9] | E627K is a well characterized determinant of increased polymerase activity in mammalian cell lines and increased virulence in mammals; studies reviewed in Suttie *et al*. (2019) [55] |
| PB2 674 | Host discrimination [1, 9] | None found |
| PB1 12 | Pandemic lineage assignment [40, 42] | None found |
| PB1 175 | Pandemic lineage assignment [40, 42] | None found |
| PB1 211 | Characteristics of infection [2, 47] | None found |
| PB1 375 | Human adaptability [27, 31] | None found |
| PB1 486 | Pandemic lineage assignment [40, 42] | None found |
| PB1 576 | Pandemic lineage assignment [40, 42] | None found |
| PB1 618 | Pandemic lineage assignment [40, 42] | None found |
| PB1 638 | Pandemic lineage assignment [40, 42] | None found |
| PB1 728 | Pandemic lineage assignment [40, 42] | None found |
| PA 55 | Host discrimination [2, 9, 10] | None found |
| PA 57 | Host discrimination [9, 10] | None found |
| PA 94 | Human adaptability [27, 30, 31] | None found |
| PA 394 | Human adaptability [29-31] | None found |
| HA 226^†^ | Human adaptability [27, 29] | Q226L is a well characterized determinant of enhanced binding toα2,6-linked sialic acid receptors; studies reviewed in Suttie *et al*. (2019) [55] |
| HA 257^†^ | Host discrimination [5, 9] | None found |
| HA 277^†^ | Host discrimination [5, 9] | None found |
| HA 344^†^ | Host discrimination [5, 9] | None found |
| NP 16 | Host discrimination [2, 9] | Alterations at NP16 in H1N1, H6N1, and H5N1 impacts virulence in mice and chickens [56] |
| NP 283 | Host discrimination [2, 9] | None found |
| NP 313 | Host discrimination [2, 9] | None found |
| NP 357 | Host discrimination [2, 9] | None found |
| M1 95 | Host discrimination [8, 10] | None found |
| M1 101 | Pandemic lineage assignment [40, 42] | None found |
| M1 115 | Host discrimination [8-10] | None found |
| M1 116 | Host discrimination [8, 10] | None found |
| M1 121 | Host discrimination [2, 8, 10] | None found |
| M1 137 | Pandemic lineage assignment [40, 42] | None found |
| M1 147 | Pandemic lineage assignment [40, 42] | None found |
| M1 160 | Pandemic lineage assignment [40, 42] | None found |
| M1 166 | Pandemic lineage assignment [40, 42] | None found |
| M1 167 | Host discrimination [8, 10] | None found |
| M1 239 | Host discrimination [9, 10] | None found |
| M2 11 | Host discrimination [9, 10] | None found |
| M2 14 | Host discrimination [9, 10] | None found |
| M2 16 | Host discrimination [9, 10] | None found |
| M2 18 | Host discrimination [9, 10] | None found |
| M2 20 | Host discrimination [8-10] | None found |
| M2 27 | Host discrimination [8-10] | None found |
| M2 28 | Host discrimination [8-10] | None found |
| M2 31 | Host discrimination [9, 10] | None found |
| M2 54 | Host discrimination [8-10] | None found |
| M2 55 | Host discrimination [8-10] | None found |
| M2 78 | Host discrimination [8-10] | None found |
| M2 82 | Host discrimination [9, 10] | None found |
| NS1 27 | Human adaptability [29, 30] | None found |
| NS1 70 | Host discrimination [2, 8, 9] | None found |
| NS1 87 | Host discrimination [2, 8, 9] | None found |
| NS1 91 | Host discrimination [8-10] | None found |
| NS1 114 | Human adaptability [27, 30, 31] | None found |
| NS1 125 | Host discrimination [8-10] | NS1 D125G enhanced binding toα2,6-linked sialic acid receptors and increased pathogenicity in mice for H3N2 [57] |
| NS2 40 | Host discrimination [8-10] | None found |
| NS2 63 | Host discrimination [2, 8, 9] | None found |
| ^†^H3 numbering |  |  |
|  | | |

**Figure S1. The most commonly used pre-processing approaches to represent the nucleotide or amino acid sequence of an influenza A virus isolate. A) amino acid position coded as integers, B) nucleotide position coded as integers, C) guanine-cytosine dinucleotide composition, D) one-hot encoding of amino acid sequence.**

**References**

1. Aguas R, Ferguson NM. Feature selection methods for identifying genetic determinants of host species in RNA viruses. PLoS Computational Biology **2013**; 9(10): e1003254.

2. Allen JE, Gardner SN, Vitalis EA, Slezak TR. Conserved amino acid markers from past influenza pandemic strains. BMC Microbiology **2009**; 9(1): 77.

3. Attaluri PK, Zheng X, Chen Z, Lu G. Applying machine learning techniques to classify H1N1 viral strains occurring in 2009 flu pandemic. BIOT-2009 **2009**; 21.

4. Attaluri PK, Chen Z, Lu G. Applying neural networks to classify influenza virus antigenic types and hosts. In: 2010 IEEE Symposium on Computational Intelligence in Bioinformatics and Computational Biology: IEEE, 2010:1.

5. ElHefnawi M, Sherif FF. Accurate classification and hemagglutinin amino acid signatures for influenza A virus host-origin association and subtyping. Virology **2014**; 449: 328.

6. Eng CL, Tong JC, Tan TW. Predicting host tropism of influenza A virus proteins using random forest. BMC Medical Genomics **2014**; 7 Suppl 3: S1.

7. Hu W. Characterization of Asian and North American avian H5N1. American Journal of Molecular Biology **2011**; 1: 52.

8. Hu W. Host markers and correlated mutations in the overlapping genes of influenza viruses: M1, M2; NS1, NS2; and PB1, PB1-F2. Natural Science **2010**; 2(11): 1225.

9. Hu W. Nucleotide host markers in the influenza A viruses. Journal of Biomedical Science and Engineering **2010**; 3(07): 684.

10. Kargarfard F, Sami A, Mohammadi-Dehcheshmeh M, Ebrahimie E. Novel approach for identification of influenza virus host range and zoonotic transmissible sequences by determination of host-related associative positions in viral genome segments. BMC Genomics **2016**; 17(1): 925.

11. Kincaid C. N-Gram methods for influenza host classification. In: Proceedings of the International Conference on Bioinformatics & Computational Biology: The Steering Committee of The World Congress in Computer Science, Computer Engineering and Applied Computing, 2018:105.

12. King D, Miller Z, Jones W, Hu W. Characteristic sites in the internal proteins of avian and human influenza viruses. Journal of Biomedical Science and Engineering **2010**; 3(10): 943.

13. Kwon E, Cho M, Kim H, Son HS. A study on host tropism determinants of influenza virus using machine learning. Current Bioinformatics **2020**; 15(2): 121.

14. Li H, Sun F. Comparative studies of alignment, alignment-free and SVM based approaches for predicting the hosts of viruses based on viral sequences. Scientific Reports **2018**; 8(1): 10032.

15. Shaltout N, Rafea A, Moustafa A, ElHefnawi M. Using information gain to compare the effeciency of machine learning techniques when classifying influenza based on viral hosts. Transactions on Engineering Technologies: Springer, **2015**:707.

16. Sherif FF, El Hefnawi M, Kadah Y. Genomic signatures and associative classification of the hemagglutinin protein for human versus avian versus swine influenza A viruses. In: 2011 28th National Radio Science Conference: IEEE, 2011:1.

17. Xu B, Tan Z, Li K, Jiang T, Peng Y. Predicting the host of influenza viruses based on the word vector. PeerJ **2017**; 5: e3579.

18. Yin R, Zhou X, Zheng J, Kwoh CK. Computational identification of physicochemical signatures for host tropism of influenza A virus. Journal of Bioinformatics and Computational Biology **2018**; 16(6): 1840023.

19. Yin R, Zhou X, Rashid S, Kwoh CK. HopPER: an adaptive model for probability estimation of influenza reassortment through host prediction. BMC Medical Genomics **2020**; 13(1): 9.

20. Eng CL, Tong JC, Tan TW. Distinct host tropism protein signatures to identify possible zoonotic influenza A viruses. PLoS One **2016**; 11(2): e0150173.

21. Eng CLP, Tong JC, Tan TW. Predicting zoonotic risk of influenza A viruses from host tropism protein signature using random forest. International Journal of Molecular Sciences **2017**; 18(6): 1135.

22. Kou Z, Lei F, Wang S, Zhou Y, Li T. Molecular patterns of avian influenza A viruses. Chinese Science Bulletin **2008**; 53(13): 2002.

23. Li J, Zhang S, Li B, et al. Machine learning methods for predicting human-adaptative influenza A viruses based on viral nucleotide compositions. Molecular Biology and Evolution **2019**; 37(4): 1224.

24. Qiang X, Kou Z, Fang G, Wang Y. Scoring amino acid mutations to predict avian-to-human transmission of avian influenza viruses. Molecules **2018**; 23(7): 1584.

25. Qiang X, Kou Z. Predicting interspecies transmission of avian influenza virus based on wavelet packet decomposition. Computational Biology and Chemistry **2019**; 78: 455.

26. Qiang X, Kou Z. Prediction of interspecies transmission for avian influenza A virus based on a back-propagation neural network. Mathematical and Computer Modelling **2010**; 52(11-12): 2060.

27. Qiang X, Kou Z. Scoring amino acid mutation to predict pandemic risk of avian influenza virus. BMC Bioinformatics **2019**; 20(Suppl 8): 288.

28. Scarafoni D, Telfer BA, Ricke DO, Thornton JR, Comolli J. Predicting influenza A tropism with end-to-end learning of deep networks. Health Security **2019**; 17(6): 468.

29. Sun Y, Zhang K, Qi H, et al. Computational predicting the human infectivity of H7N9 influenza viruses isolated from avian hosts. Transboundary and Emerging Diseases **2020**; 00: 1.

30. Wang J, Kou Z, Duan M, Ma C, Zhou Y. Using amino acid factor scores to predict avian-to-human transmission of avian influenza viruses: a machine learning study. Protein & Peptide Letters **2013**; 20(10): 1115.

31. Wang J, Ma C, Kou Z, Zhou YH, Liu HL. Predicting transmission of avian influenza A viruses from avian to human by using informative physicochemical properties. International Journal of Data Mining and Bioinformatics **2013**; 7(2): 166.

32. Wang J, Zhou Y, Kou Z. One class support vector machine for predicting avian-to-human transmission of avian influenza A virus. In: 2012 IEEE International Conference on Computer Science and Automation Engineering: IEEE, 2012:184.

33. Attaluri PK, Chen Z, Weerakoon AM, Lu G. Integrating decision tree and Hidden Markov Model (HMM) for subtype prediction of human influenza A virus. In: International Conference on Multiple Criteria Decision Making: Springer, 2009:52.

34. Chrysostomou C, Seker H. Signal-processing-based bioinformatics approach for the identification of influenza A virus subtypes in neuraminidase genes. Conference Proceedings - IEEE Engineering in Medicine and Biology Society **2013**; 2013: 3066.

35. Ebrahimi M, Aghagolzadeh P, Shamabadi N, et al. Understanding the undelaying mechanism of HA-subtyping in the level of physic-chemical characteristics of protein. PLoS One **2014**; 9(5): e96984.

36. Fabijańska A, Grabowski S. Viral genome deep classifier. IEEE Access **2019**; 7: 81297.

37. Humayun F, Khan F, Fawad N, et al. Computational Method for Classification of Avian Influenza A Virus Using DNA Sequence Information and Physicochemical Properties. Frontiers in Genetics **2021**; 12: 10.

38. Shepard SS, Davis CT, Bahl J, Rivailler P, York IA, Donis RO. LABEL: fast and accurate lineage assignment with assessment of H5N1 and H9N2 influenza A hemagglutinins. PLoS One **2014**; 9(1): e86921.

39. Wang Y, Bao J, Du J, Li Y. Rapid detection and prediction of influenza A subtype using deep convolutional neural network based ensemble learning. In: Proceedings of the 2020 10th International Conference on Bioscience, Biochemistry and Bioinformatics, 2020:47.

40. Hu W. Novel host markers in the 2009 pandemic H1N1 influenza A virus. Journal of Biomedical Science and Engineering **2010**; 3(06): 584.

41. Kargarfard F, Sami A, Ebrahimie E. Knowledge discovery and sequence-based prediction of pandemic influenza using an integrated classification and association rule mining (CBA) algorithm. Journal of Biomedical Informatics **2015**; 57: 181.

42. Kargarfard F, Sami A, Hemmatzadeh F, Ebrahimie E. Identifying mutation positions in all segments of influenza genome enables better differentiation between pandemic and seasonal strains. Gene **2019**; 697: 78.

43. Meroz D, Yoon SW, Ducatez MF, et al. Putative amino acid determinants of the emergence of the 2009 influenza A (H1N1) virus in the human population. Proceedings of the National Academy of Sciences of the United States of America **2011**; 108(33): 13522.

44. Chadha A, Dara R, Poljak Z. Convolutional classification of pathogenicity in H5 avian influenza strains. In: 2019 18th IEEE International Conference On Machine Learning And Applications: IEEE, 2019:1570.

45. Ivan FX, Kwoh CK. Rule-based meta-analysis reveals the major role of PB2 in influencing influenza A virus virulence in mice. BMC Genomics **2019**; 20(Suppl 9): 973.

46. Long GS, Hussen M, Dench J, Aris-Brosou S. Identifying genetic determinants of complex phenotypes from whole genome sequence data. BMC Genomics **2019**; 20(1): 470.

47. Peng Y, Zhu W, Feng Z, et al. Identification of genome-wide nucleotide sites associated with mammalian virulence in influenza A viruses. Biosafety and Health **2020**; 2(1): 32.

48. Shaltout N, Moustafa M, Rafea A, Moustafa A, ElHefnawi M. Comparing PCA to information gain as a feature selection method for Influenza-A classification. In: 2015 International Conference on Intelligent Informatics and Biomedical Sciences (ICIIBMS): IEEE, 2015:279.

49. Shaltout N, Rafea A, Moustafa A, Moustafa M, ElHefnawi M. Optimizing the detection of antiviral-resistant influenza-A strains using machine learning. In: Proceedings of the World Congress on Engineering and Computer Science, 2016:1.

50. Kim JH, Hatta M, Watanabe S, Neumann G, Watanabe T, Kawaoka Y. Role of host-specific amino acids in the pathogenicity of avian H5N1 influenza viruses in mice. The Journal of General Virology **2010**; 91(Pt 5): 1284.

51. Bussey KA, Bousse TL, Desmet EA, Kim B, Takimoto T. PB2 residue 271 plays a key role in enhanced polymerase activity of influenza A viruses in mammalian host cells. Journal of Virology **2010**; 84(9): 4395.

52. Foeglein A, Loucaides EM, Mura M, Wise HM, Barclay WS, Digard P. Influence of PB2 host-range determinants on the intranuclear mobility of the influenza A virus polymerase. The Journal of General Virology **2011**; 92(Pt 7): 1650.

53. Mok CKP, Lee HHY, Lestra M, et al. Amino acid substitutions in polymerase basic protein 2 gene contribute to the pathogenicity of the novel A/H7N9 influenza virus in mammalian hosts. Journal of Virology **2014**; 88(6): 3568.

54. Xiao C, Ma W, Sun N, et al. PB2-588 V promotes the mammalian adaptation of H10N8, H7N9 and H9N2 avian influenza viruses. Scientific Reports **2016**; 6: 19474.

55. Suttie A, Deng Y-M, Greenhill AR, Dussart P, Horwood PF, Karlsson EA. Inventory of molecular markers affecting biological characteristics of avian influenza A viruses. Virus Genes **2019**; 55(6): 739.

56. Lipatov A, Yen H-L, Salomon R, Ozaki H, Hoffmann E, Webster R. The role of the N-terminal caspase cleavage site in the nucleoprotein of influenza A virus in vitro and in vivo. Archives of Virology **2008**; 153(3): 427.

57. Narasaraju T, Sim M, Ng H, et al. Adaptation of human influenza H3N2 virus in a mouse pneumonitis model: insights into viral virulence, tissue tropism and host pathogenesis. Microbes and Infection **2009**; 11(1): 2.
